# Poplar Hospital for Accidents

**Published:** 1902-04-05

**Authors:** 


### POPLAR HOSPITAL FOR ACCIDENTS.

The annual general meeting of governors on Tuesday, March 25th, was preceded by a special meeting to alter one of the rules. This was very quickly done, the matter, as explained by the Chairman, the Hon. Sydney Holland, being a simple one. The delay and expense consequent on a change of trustees, owing to death or any other cause, has been recently avoided at Poplar Hospital by placing all investments in the hands of the Law Guarantee and Trust Society, and the alteration merely ensures the continuance of present investments *in statu quo*, and refers, of course, only to investments which have been transferred to the Society, and not to those which they may make in the future.

This over, Mr. Holland addressed the meeting, which consisted of about 16 men, most of them, to quote the report, of those who "toil and spin." On the platform was the secretary and house governor, Lieut.-Colonel Feneran, while Mr. J. E. Broodbank, vice-chairman, was also present.

### Phenomenal Success.

The year, said Mr. Holland, had been a record one as to receipts, extension, and the number of patients treated. He did not suppose it would ever be exceeded; however, that had been said before. For a little hospital the results were splendid, and it was owing to Mr. Edgar Speyer that the receipts had been so high. Mr. Speyer presided at the annual dinner, and he not only gave £500 himself, but managed to interest a great number of people in the hospital, so much so that his own list amounted to no less than £4,030. The new children's ward had been called after him, in recognition of this. Three beds had been dedicated to the memory of three members of the Domville family; Miss Domville, a constant visitor since 1891, had given £3,015 for this purpose. He was glad to say that the reserve fund had been added to. It was very necessary to be prepared for a rainy day. It was hoped that at least £20,000 might be put by in this way. The work of the hospital was not going to be extended in any way until that very reasonable sum was ready. The total receipts towards upkeep had been £11,802, and £3,392 had been received towards the building and furnishing fund. In legacies £687 had been received. The sum of £7,865 had been paid as ordinary expenditure; £7,882 for building; £2,946 had been invested; and £2,956 was carried forward. Of this, however, £2,400 was owing to the builders. The Hospital Saturday Fund had asked them to have a representative on their committee; he would be gladly welcomed. He thought the results showed that if those responsible for a hospital did their very best to make it as perfect as possible, people would help. If Poplar Hospital had been left to drift, as it was doing ten or twelve years ago, it would not have been what they saw it to-day. A satisfactory item on the balance-sheet was the payment from the out-patients for bandages. Some of the working men regarded it at one time with disfavour, but at two large meetings it was agreed to, and as the payments were only made by those who could afford it, and who wished to do so, there did not appear to be any hardship. Frequently, too, more was actually paid than the twopence asked for. He had seen an article in the *Times* the previous day recommending the plan.

### The Saving to the Rates.

Mr. J. E. Broodbank, vice-chairman, drew attention to the saving to the local rates effected by this hospital, namely, sixpence in the pound. He added that, if the local authorities had done last year what this hospital did, it would have added 1s. 3d. in the pound to the rates.

### A Paying Ward.

Two of the wards added to the hospital last June, when the Master of the Drapers' Company and the Bishop of London opened the new medical wing, have been since fully occupied; one, however, has not yet been opened. The cost of maintaining it is estimated at £700, and, as is well known, the Poplar Hospital's policy is *never to be in debt*. Mr. Holland explained to the meeting that when the Drapers' Company gave £14,000 for the building of the new wing only two wards were contemplated. It was found, however, that the work could be done far more cheaply if three were added, but up till now only two had been opened. The committee, realising the cost of opening the third on the same terms, had settled to open it as a ward for paying patients. Mr. Holland went on to point out that there were numbers of clerks with whom it would go very much against the grain to come in free, but who could not afford to pay full doctors' fees and the expense of a private nurse. To a man earning, say, £200 a year it was quite as great a charity to provide medical treatment for a sum which he could afford to pay as it was to give it free to those who could afford to contribute nothing towards the expense. "They are quite as much in need of good nursing," said Mr. Holland, "as we are. Hereafter, if the hospital gets very rich, we may think it is better to return to having them all free, but for the present that is the committee's decision. Do you agree?"

"Agreed," the meeting replied.

Mr. Broodbank asked whether a parapraph on the matter could be inserted into the report, which, in its draft form, made no allusion to the committee's decision. The Chairman replied that the decision had been made during the present year, but that he thought it might certainly be mentioned.

### A Tribute to the Staff.

The rest of the time was occupied in the more formal business. Mr. Piper, in moving a vote of thanks to the staff, said he often came to visit patients in the hospital, sometimes in the middle of the night, and "there they are," he said, "to their duty. I'm sure the patients would get the attention the matron and nurses give in no other hospital. In our matron we have a gem, and no mistake." Alluding to the resignation of the chaplain, the Rev. A. N. Campbell, who, though blind, ministered for 11 years without fee or reward, Mr. Piper said he had not the pleasure of knowing the new chaplain, but they must learn to love him as they had the old one.

After the meeting tea, presided over by Miss Bland, the matron, and the home sister, was provided in the doctors' room, and an informal discussion took place on the selection of a chairman for the dinner. Several of the governors took the opportunity of visiting the new wards and nurses' rooms, all of which are very bright and cheerful, the latter being furnished in excellent taste. "I think," said one of the committee, "that if ladies give themselves to a hospital, they ought to have a nice place to come to when they're off duty."

